# Combining methods to conduct a systematic review and propose a conceptual and theoretical framework in socio-environmental research

**DOI:** 10.1016/j.mex.2023.102484

**Published:** 2024-01-05

**Authors:** Indira A. L. Eyzaguirre, Marcus E. B. Fernandes

**Affiliations:** a Programa de Pós-graduação em Biologia Ambiental, Laboratório de Ecologia de Manguezal (LAMA), Instituto de Estudos Costeiros (IECOS), Universidade Federal do Pará (UFPA), Alameda Leandro Ribeiro, CEP 68600-000, Aldeia, Bragança, Pará, Brazil; b Sarambui Civil Society Organization, Bragança, Pará, Brazil; c Departamento de Pesquisa, Resiliencia Innovadora, Lima, Perú

**Keywords:** SODIP steps 1. Studies of systematic review and meta-analyses. 2. Open-source (software and data) use. 3. Data visualization and design information. 4. Identifying gaps, challenges and trends. 5. Propose a conceptual and theoretical framework., Systematic review, Bibliometric, Meta-analysis, SODIP steps, Open-source tools, Theoretical framework, Conceptual framework, Mangrove

## Abstract

This study aims to present a combination of methods and propose robust theoretical and conceptual frameworks for solving socio-environmental issues. This proposal included the Problem, Intervention, Context, and Outcome (PICO) framework and Protocol and Reporting result with Search, Appraisal, Synthesis, and Analysis framework, and develop (PSALSAR) method through SODIP steps: (i) Systematic review and meta-analysis defining the study from guiding questions; (ii) Open-source related to software and data; (iii) Data visualization and design information; (iv) Identification of gaps, challenges and trends through automation and lexicometric analysis; and (v) Proposal of theoretical and conceptual frameworks. This proposal defines the steps as support to combine and systematize information necessary to facilitate the production of this type of document using open-access software in the visualization and design of information. All these steps are replicable and essential to propose a conceptual and theoretical framework to contribute to the construction of knowledge in socio-environmental research and to propose solutions by filling in the gaps.

In summary, this combination of methods shows:

• The use of SODIP steps provides robustness and efficiency in carrying out review studies, facilitating the way to propose theoretical or conceptual frameworks.
• Choosing to use open-source tools is essential for better evaluation and visualization of qualitative and quantitative data in review studies.
• The combination of methods and data in the systematic review (scientific, political documents. and databases) supports the proposal of robust theoretical and conceptual frameworks.

## Highlights

• SODIP steps provide robustness and efficiency for theoretical/conceptual framework.
• Systematic review articles combined with georeferenced data support the framework.
• Combination of scientific and policy data supports theoretical/conceptual framework.
• Using open-source tools is essential for better evaluation and visualization of data.

## Graphical abstract

**Figure:**
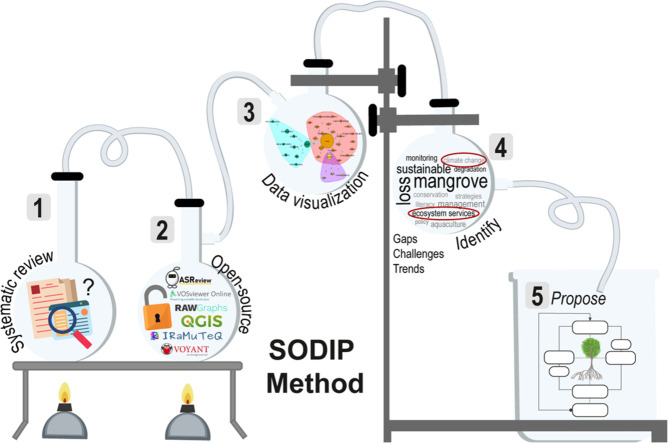

Specifications Table

**Table:** 

Subject Area:	Environmental Science
More specific subject area:	Environmental science
Method name:	SODIP steps 1. Studies of systematic review and meta-analyses 2. Open-source (software and data) use 3. Data visualization and design information 4. Identifying gaps, challenges and trends 5. Propose a conceptual and theoretical framework
Name and reference of original method:	I.A.L. Eyzaguirre, A.Y. Iwama, M.E.B. Fernandes, Integrating a conceptual framework for the sustainable development goals in the mangrove ecosystem: A systematic review, Environmental Development. 47 (2023) 100,895. 10.1016/j.envdev.2023.100895.
Resource availability:	*Eyzaguirre, Indira Angela (2023), “SODIP steps”, Mendeley Data, V1, doi: 10.17632/c8br5yzz4f.1* https://data.mendeley.com/datasets/c8br5yzz4f/1

## Method details

Review studies respond robustly to different issues and go beyond a bibliometric analysis, which statistically evaluates the productivity performance of a given research group [1,2]. These studies can be compared to experiments carried out in laboratories, considering data management from collection to visualization. Socio-environmental research, for example, is constantly changing as it needs a holistic view to address its issues [3]. These socio-environmental studies involve all areas of research, from environmental, innovation and technology to social sciences, as complex issues arise where solutions also need to be innovative. In this way, the review studies used to propose a conceptual and theoretical framework are a powerful tool for identifying gaps, challenges, and trends, as this involves planning, identifying, mapping, collecting, systematizing, reporting and visualizing data (theoretical and practical) over a given period of time [4,5]. Here, we present the SODIP methodology that proposes 5 steps to propose a framework based on systematic review studies combined with open access data, according to the needs of the study.

## SODIP step 1: systematic review and meta-analysis

### Defining the systematic review study

**Fig. 1:**
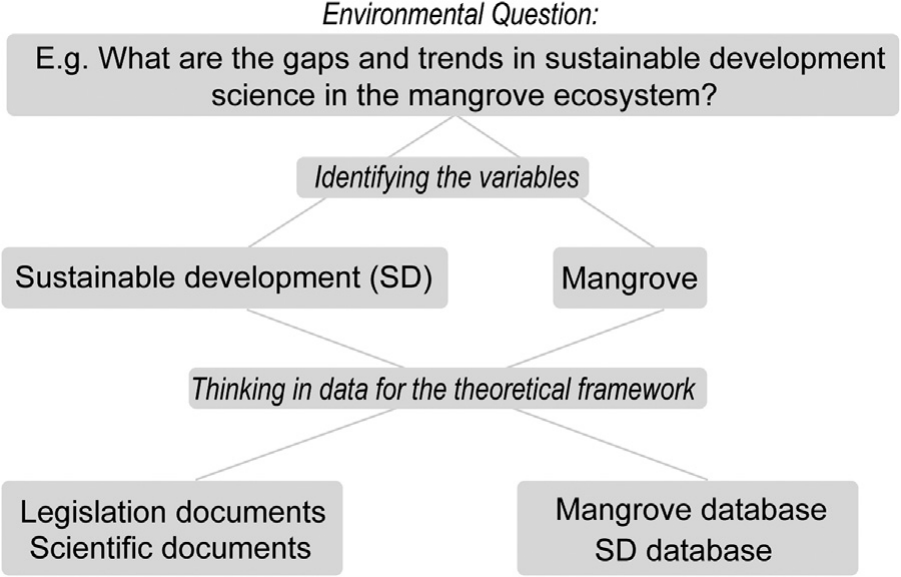
Example to define the question and variables of interest for systematic review studies. Based on [4].

Basic steps of planning to conduct a review study are necessary. Guiding questions must be answered, mainly to identify the topic addressed and define the proposed theoretical framework (Table 1). To define the questions, the cognitive map [6](Siau and Tan, 2005) can be used, as it facilitates the process of identifying the problem and its questions that will help to design the research protocol (Fig. 1). The protocol must be elaborated, database searches conducted, articles screened, extraction and critical analysis of data performed, data synthesized, the report created and finalized review performed [7]. The definition of a review protocol is an extremely relevant step, as it promotes coherence, integrity and transparency in this type of study [8].

**Table 1:** Objective Definition [7,9].

Question for systematic review and theoretical framework	Objective
What is the theory of the topic to be studied?	Identify the main theme
What are the variables of interest?	Identify the variables of interest
	Fill in the gaps
What are the main gaps in knowledge?	Identify the main challenges in the topic addressed
What are the main challenges of this topic?	Define the main topics covered
What are the trends in studies on this topic?	Identify the main trends and gaps to define the framework

An important step in review studies is the definition of objectives, which must be qualitatively (data visualization) or quantitatively (statistical analysis) measurable (Table 2). The objectives define the direction that the systematic review will take, whether it is a critical analysis of published research, addresses methodologies used for a given topic or an exploratory assessment to present research evidence.

**Table 2:** Examples of objectives in review studies.

Title	Objective
Beyond PRISMA: Systematic reviews to inform marine science and policy	“We identified eighteen systematic reviews published on marine topics between 2008 and 2015″ [10]
A review of remote sensing for mangrove forests: 1956–2018	“The objectives of this study are: 1) to identify key milestones of RS of mangrove forests to provide a historical overview of this research field in the chronological order; 2) to discover key drivers for the evolution of different milestones to analyze theoretical developments of mangrove RS, and 3) to project future research directions in mangrove RS.” [11]
Review of valuation methods for mangrove ecosystem services	“We present a comprehensive overview and summary of studies undertaken to investigate the ecosystem services of mangrove forests. We address the variety of different methods applied for different ecosystem services evaluation of mangrove forests, as well as the methods and techniques employed for data analyses, and further discuss their potential and limitations." [12]
The costs and benefits of REDD+: A review of the literature	“We conducted a review of 60 unique REDD+ costs and benefits studies.” [13]
Systematic Review of Spatial Planning and Marine Protected Areas: A Brazilian Perspective	“This article offers a systematic review through a comparative meta-analysis of the literature on MPAs and spatial planning. Specific goals of this study are (I) to identify studies on Brazil; and (II) to compare and contrast these with studies performed elsewhere.” [14]
Analyzing 70 years of research output on South African estuaries using bibliometric indicators	“This study examined scientific papers published in authoritative international journals authored by researchers working on South African estuaries between 1949 and 2020″ [15]
Brazilian Mangroves: Blue Carbon Hotspots of National and Global Relevance to Natural Climate Solutions	“We provide a direct comparison between mangroves and Brazil's other major vegetated biomes, identifying mangroves as a major carbon hotspot that can help meet Intended Nationally Determined Contributions (NDCs), in addition to their significance as global coastal carbon sinks." [16]
Integrating a conceptual framework for the sustainable development goals in the mangrove ecosystem: A systematic review	“The present study aimed to propose a conceptual framework for SDG in relation to the mangrove ecosystem, as a baseline scenario, based on a systematic review relating them to Ramsar Sites.”[4]

Once the questions (issue) and objectives of the review study have been defined, it is necessary to define the timeline or time scale of the study to be used to elaborate the protocol.

### Protocol search

The protocol describes the methodology used step-by-step to conduct review studies. Para elaborar o protocol é necessário definir o contexto do estudo de revisão, desde a escala temporal, a delimitação espacial, palavras-chave, os critérios de inclusão e exclusão e as bases de dados a serem consultadas.The definition of topics was based on the Protocol Search Appraisal Synthesis Analysis Report (PSALSAR) (Table 3) and Population Intervention, Comparison, and Outcomes (PICOC) framework (Table 4) [5] in order to identifying evidence needs.

**Table 3:** PSALSAR Framework of the systematic review studies.

Steps	Objective	Outcomes
Protocol	Define the study scope depending on the scale (global, transnational, national or local)	Examples: • Analyzing 70 years of research output on South African estuaries using bibliometric indicators • Systematic Review of Spatial Planning and Marine Protected Areas: A Brazilian Perspective
Search	Define the search method	Plan the databases accessible to the researcher thinking about the effectiveness of the search [7,[17], [18], [19]].
Appraisal	Selection of documents (scientific, legislation) and/or critical data	Define inclusion and exclusion criteria. Example: • Table 1 of Systematic Review of Spatial Planning and Marine Protected Areas: A Brazilian Perspective
Synthesis	Categorization of documents and/or data	Using encoding manually or through software. This coding will facilitate the quantitative analysis and visualization of the data. Example: • Spatial scale: Global (G), Transnational (T), National (N) and Local (L) • Ecosystem services according to CICES: Regulation and maintenance (RM), Provisioning services (PS) and Cultural services (CS)
Analysis	Data analysis and visualization	The previous step (coding) will support data analysis and visualization, because if the database is organized, it is easier to generate interesting graphics that represent documents and/or data.
Report	Conclusion and report on the final production	In this step, PRISMA is often used, although this reporting methodology is used in the screening of the data, it is also a reference to carry out more accurately [8].

Modified from Mengist et al. (2020).

**Table 4:** PICO framework of the systematic review studies.

Concept	PIPOC Framework
Population	Define the theme including the ecosystem or socio-ecological system addressed. Examples: Which countries have a mangrove ecosystem? What are the Marine Protected Areas that conserve mangroves? What are the Ramsar Sites that harbor mangroves?
Intervention	Identify the methodologies, methods and tools that best assess the topic addressed. Example: Identify the direct and indirect drivers that impact the mangrove List the indicators addressed to better assess the impacts on the mangrove Identify the valuation methodologies of mangrove ecosystem services
Comparison	Comparison of topics covered, methods, indicators or other topics within the same studies and between them. Example: Methodologies that best assess the impacts on mangroves GIS and RS methodological approaches to assessing impacts on mangroves
Outcomes	Factors in studies on the topic are addressed in the ecosystem according to spatial scale. Example: Valuation indicators of mangrove ecosystem services around the world
Context	Identify challenges and obstacles in research on the topic addressed in the related ecosystem. Example: Methodologies applied to specific contexts on mangrove conservation

The essential steps of the protocol in review studies are: (i) selection of temporal scale; (ii) selection of spatial scale; (iii) selection of search words; (iv) selection of search operators; (v) selection of search sections; (vi) selection of databases for scientific documents; and (vii) inclusion and exclusion criteria.

i. Selection of temporal scale through the search dates are an important point. An important point to be raised and which is not common in review studies is the identification of international events and treaties according to the topic covered. This can be used as a baseline to define the time scale. In the same time, the database or criteria defined by the researcher based on a relevant factor such as an international treaty (e.g., Ramsar Convention) or important date (e.g., the definition of the 2030 Agenda and the SDGs) (Table 5).

**Table 5:** Important dates for the mangrove ecosystem and socio-environmental research.

Description	Year
Ramsar Convention on Wetlands	1971
1st RAMSAR site in mangrove	1974
1st World Climate Conference	1979
1st IPCC report	1990
UNFCCC	1994
1st COP	1996
Kyoto Protocol	2005
Copenhagen Accord	2009
Paris Agreement	2015
2030 Agenda and SDG	2015
Decade of Action	2020
Ocean Decade	2021
ii. Selection of spatial scale or multiple scales (Fig. 2) is related to the type of review study being conducted: (i) Global, (ii) Transnational, both appropriate for Earth System Science, International Law, and International Relations, (iii) National, (iv) Regional, both appropriate for global sustainability science (law, sociology, political science, geography, and development studies), (v) Community, and (vi) Individual [20].

**Fig. 2:**
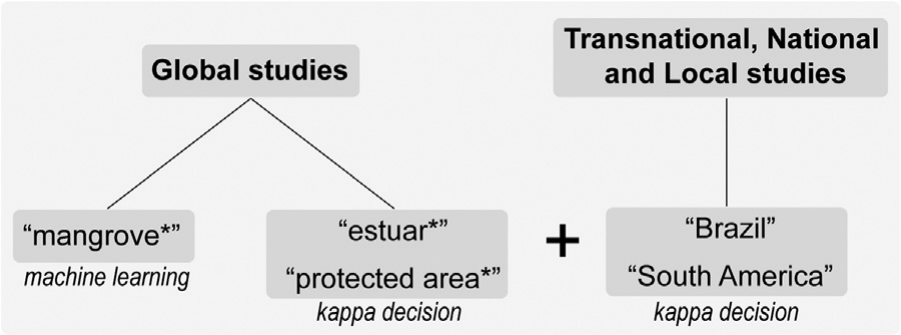
Example of search words according to the spatial scale of the review studies.
iii. The selection of search words must be carefully chosen, in two ways depending on the spatial scale of the review study as this will define the type of analysis. To obtain greater precision in the search, the words must be combined [18]. Abbreviations can be used, as long as they are found in the literature (e.g., SDG OR SDGs referring to Sustainable Development Goals) or even compound words (e.g., Land use OR Land-use). Some encodings must be used for the search to be more accurate and efficient (Table 6).

**Table 6:** Examples of coding search words.

Codification	Description	Example
“ ”	To find the word exactly without variations	“mangrove” “estuarine”
*	To find the word and its derivations in singular and plural	“mangrove*” “estuar*”

See more in WoS: https://webofscience.help.clarivate.com/en-us/Content/search-operators.html
iv. Selection of databases for scientific documents: The selection of databases must be chosen carefully (Table 6). Some articles present evidence and discrepancies between the databases for commonly used review studies, especially regarding their efficiency [19,[21], [22], [23]]. Some of the most common databases are: (i) Web of Science (WoS), (ii) Scopus, (iii) Scielo, and (iv) Google Scholar.
v. Selection of search operators: In this step, the Boolean operators are selected and everything will depend on the scope of the studies that will be selected in the review study (Table 7).

**Table 7:** Boolean Operator Search Samples.

Search words	Section	WoS	Scopus	Google scholar
“mangrove*”	Title	11,971	14,380	746,000
	Abstract	17,281	23,722	
	Keywords	7978	16,262	
	Title, abstract, keywords	20,022	26,300	
“mangrove*” AND “sustainable development goal*”	Title	2	2	1510
	Abstract	29	41	
	Keywords	3	15	
	Title, abstract, keywords	29	53	
“mangrove*” OR “sustainable development goal*”	Title	14,765	17,722	1270,000
	Abstract	29,505	39,413	
	Keywords	12,191	22,955	
	Title, abstract, keywords	34,293	44,625	

Example of a search formula in WoS: (TI=(“mangrove*” OR “sustainable development goal*”)) OR (AB=(“mangrove*” OR “sustainable development goal*”)) OR (AK=(“mangrove*” OR “sustainable development goal*”)). See more in WoS: https://webofscience.help.clarivate.com/en-us/Content/search-operators.html#Search.

https://images.webofknowledge.com/images/help/WOS/hs_advanced_fieldtags.html.

http://schema.elsevier.com/dtds/document/bkapi/search/SCOPUSSearchTips.htm.
vi. Selection of search sections: Search sections refer to sections in the document (e.g., title, abstract, keywords) and other components (e.g., funding, author). Although there is a wide range of sections to choose from, “Title”, “Abstract” and “Keywords” are the most efficient [24] (see Table 7).See more in WoS and Scopus:https://images.webofknowledge.com/images/help/WOS/hs_advanced_fieldtags.html
  http://schema.elsevier.com/dtds/document/bkapi/search/SCOPUSSearchTips.htm
  The better definition of these items provides more robustness to the search and greater data precision, in accordance with the defined objectives. Some examples are presented in Table 8.

**Table 8:** Examples of review studies.

Spatial scale	Title	Temporal scale	Search words	Database
Global studies	Beyond PRISMA: Systematic reviews to inform marine science and policy	2008–2015	–	Web of Science, Scopus and Google Scholar i
	A review of remote sensing for mangrove forests: 1956–2018	1956–2018	-	-
	The costs and benefits of REDD+: A review of the literature	1995–2015	REDD cost, REDD benefit, REDD economics, REDD financing, forest carbon cost, forest carbon benefit, forest carbon economy, forest carbon financing, the opportunity cost of deforestation, cost/benefit of preventing deforestation, and cost/benefit of tropical land conservation	Google Scholar, EconLit, Science Direct, Willey Online Library, Web of Science, and Scopus
	Integrating a conceptual framework for the sustainable development goals in the mangrove ecosystem: A systematic review	1945–2021	Sustainable development’, ‘sustainable Development Goal*’, ‘SDG’ and ‘Ramsar*’ combined with ‘mangrove*’	Web of Science (WoS) and Scopus
Transnational level	Systematic Review of Spatial Planning and Marine Protected Areas: A Brazilian Perspective	2003–2017	(I) {{“protected area*” AND “(coastal OR marine)”}, OR MPA} AND {“spatial management” OR “spatial planning”}} and (II) {{“protected area*” AND “(coastal OR marine)”}, OR {“MPA” OR “conservation unit*”^6^} AND {“spatial management” OR “spatial planning”} AND Brazil}	Web of Science (WoS) database (Clarivate Analytics, 2017) was used to search for studies outside and inside Brazil and the Brazilian Digital Library of Theses and Dissertations (BDTD)
National level	Analyzing 70 years of research output on South African estuaries using bibliometric indicators	1990–2020	“TS=(Estuar* AND South Africa*)”	WoS online databases namely SCI EXPANDED, SSCI, A&HCI, CPCI–S, CPCISSH, BKCI–S, BKCI-SSH, ESCI, CCR EXPANDED, and IC Scopus
	Brazilian Mangroves: Blue Carbon Hotspots of National and Global Relevance to Natural Climate Solutions		“carbon sequestration,” “carbon accumulation,” “wood production,” “biomass production,” “stem growth,” “basal area increment,” and “DBH increment" are always in combination with the terms "mangrove" and "Brazil." Portuguese terms "carbono" (for carbon) and "mangue*" (for mangrove or mangal)	Google Scholar, Science Direct, Web of Science, and the Brazilian SciELO
vii. The inclusion and exclusion criteria support selecting documents and improving the filtering of them. Therefore, it is extremely important to define them in accordance with the previous steps (Table 10).
viii. Selection of databases for other types of information: Selection of other databases will depend on the purpose of the review study, whether it will include data on ecology, spatial issues, legislation documents, historical data, etc. Although there are few databases on global policies, it is important to include legislation documents and unify them with scientific data [25]. In Table 9 presents some databases on socio-environmental issues.

**Table 9:** Open access databases.

Database	Description	Site
Mangrove countries [26,27]	Spatial information about mangroves around the world.	https://globil-panda.opendata.arcgis.com/items/c3522b68c37c41b78f4c1c48f5a37159
Ramsar Sites	Information about Ramsar Sites	https://rsis.ramsar.org/
Protected areas	Information about protected areas	https://www.protectedplanet.net/en
Blue solutions	About blue projects and actions	https://bluesolutions.info/
SDG portal	Statistical information about the SDGs	https://unstats.un.org/sdgs/unsdg/ https://unstats.un.org/sdgs/metadata/ http://data.uis.unesco.org/?ReportId=163
Conservation Evidence	Information on conservation evidence methodologies	https://www.conservationevidence.com/
Global fishing watch	Information on trade, fisheries and marine resources	https://globalfishingwatch.org/map-and-data/
Marine protection atlas	Database including worldwide reports	https://mpatlas.org/
OECD Data	Information about the economy	https://data.oecd.org/
FAO data	Diverse information on FAO reports, including SDG and its targets	https://www.fao.org/statistics/en/ https://www.fao.org/fishery/en/statistics
Constitute project	Information about the constitutions in the countries, making comparisons possible	https://www.constituteproject.org/
Ecolex	Database of legislation documents, treaties and political decisions	https://www.ecolex.org/
Natural Earth Data	world spatial information	https://www.naturalearthdata.com/downloads/
UN Biodiversity Lab	Provide spatial and open-access information	https://map.unbiodiversitylab.org/earth
Trends.Earth	A free access platform for data with information on some SDGs and their indicators	https://trends.earth/
Global Human Settlement (GHS)	Information about human settlement	https://ghsl.jrc.ec.europa.eu/
WorldBank	Information on various world indicators	https://data.worldbank.org/?name_desc=false
United Nations		https://dataunodc.un.org/
Our World in Data	Miscellaneous information	https://ourworldindata.org/
Toolbox for environmental data journalist (Caixa de ferramentas para jornalistas de dados ambientais)	Big collection of open databases	https://escoladedados.org/tutoriais/caixa-de-ferramentas-para-jornalistas-de-dados-ambientais/ https://docs.google.com/spreadsheets/d/18rtqh8EG2q1xBo2cLNyhIDuK9jrPGwYr9DI2UncoqJQ/edit#gid=2009597506

**Table 10:** Inclusion and exclusion criteria.

Criteria	Description	Example
Spatial scale	Review studies with location or spatial scale criteria.	• Studies in the mangroves of South America • Studies around the world • Studies in a particular country
Temporal scale	If the study is intended to compare before to after an important date.	• Studies from the Ramsar Convention (1971) • Studies after the declaration of the 2030 Agenda and the SDGs (2015)
Section	Sections such as title, abstract and Keywords can be criteria for the most efficient search.	• Scientific documents in Title search only (see the difference in Table 7)
Language	This criterion will depend on the spatial scale of the studies and also defines the local databases.	• Local studies must follow searches in the language of the country combined with English: Brazil – Portuguese, Peru – Spanish. • Local studies with local databases: Systematic Review of Spatial Planning and Marine Protected Areas: A Brazilian Perspective [14].
Type of document	There are several types of documents in the databases; their selection is a relevant criterion to take into account.	• Scientific articles to systematize methodologies and case studies • Review articles to identify knowledge gaps • Conference paper to evaluate results of important academic conferences
Peer review	This item will depend on the access the author has to the databases. In case of lack of incentive for science in the countries, including open-access documents as a criterion.	• Open access scientific documents • Access through national research platforms such as Portal Capes in Brazil • Access through platforms such as ResearchGate

### Review reporting

PRISMA (Preferred Reporting Items for Systematic Reviews and Meta-Analyses) is a report that helps to document the *a priori* route of the systematic review [8]. The review reporting consists of two stages [28]: (i) the procedure using the PRIS report and (ii) the description of the results from the applied presentation MA. To perform this report, there are predefined spreadsheets in the following formats:

a. PRISMA flow diagram: http://www.prisma-statement.org/PRISMAStatement/FlowDiagram
b. PRISMA to create a flow diagram online: https://estech.shinyapps.io/prisma_flowdiagram/
c. PRISMA in R package [29]: https://estech.shinyapps.io/prisma_flowdiagram/

### Kappa coefficient (Kw) for validation

The Cohen's kappa coefficient (Kw) statistic is used to calculate the level of agreement of selected documents between reviewers [7]. This statistical test is based on an agreement matrix [30,31]. Validation using the Kappa weighting method is a way of providing reliability to review studies [32]. To carry out the selection of documents, manually, through Kw, authors must define the main criteria for selection. For example: (i) studies that directly mention the search words in the title, (ii) studies that use innovative methodologies, (iii) studies that are within the spatial scale of the scope of the systematic review study. After that, the agreement matrix is built according to the format required by the software that will be used (see example in Table 12).

**Table 11:** List of software for statistical analysis.

Software	Description	Site
R Study	An open-source software-based programming language for data analysis and visualization [34]	https://www.rstudio.com/
Python	An open-source software for data processing [35]	https://www.python.org/
ASReview [36]	For carrying out systematic reviews manually, assisted by machine learning.	https://asreview.nl/
Power BI [37]	Software that can be used in free mode for data visualization; although it is paid, in the free modality it offers interesting visualization tools.	https://powerbi.microsoft.com/en-gb/
Xlstat [38]	One of the most powerful software programs, it can be used in the test version as a student.	https://www.xlstat.com/en/download
RawGraphs	An open-source tool for creating data visualization that comes from graphic design [39].	https://rawgraphs.io/
Flourish	Tool for visualization with many templates.	https://flourish.studio/
Looker Studio overview	A web-based data visualization tool for make dashboards.	https://datastudio.withgoogle.com/
VOSviewer 1.6.9	Constructing and visualizing scientific landscapes and bibliometric networks by Leiden University [40]	https://www.vosviewer.com/
CinET Explorer	A software that analyzes and visualizes scientific documents, especially citations [41,42].	https://www.citnetexplorer.nl/
Citespace	A tool that uses the Java language to visualize and analyze trends in scientific documents over time [43].	http://cluster.cis.drexel.edu/~cchen/citespace/">
Scimat	An open-source tool that develops analysis based on scientific mapping [44].	https://sci2s.ugr.es/scimat/
Text Analyzer	An online tool for textual linguistic analysis [45].	https://www.online-utility.org/text/analyzer.jsp
IRAMUTEQ	A program that aims to analyze the qualitative data quantitatively through descriptive and inferential statistics [46]	http://www.iramuteq.org/
Anthropac	An open software for cultural domain analysis [47]	http://www.analytictech.com/anthropac/anthropac.htm
QGis	An open-source software for analyzing and visualizing spatial data [48] that can be used for the representation of spatial data in review studies.	https://qgis.org/en/site/
Google Earth Engine (GEE)	An tool for spatial analyse and visualization [49]	https://earthengine.google.com/
Voyant tool	An open-source for visualization, mining, and analysis of text trends [50,51]	https://voyant-tools.org/
Inkscape	A tool for draw and graphic design.	https://inkscape.org/

**Table 12:** Concordance matrix.

Kappa decision		Reviewer A		Total
		Rejected	Accepted	
Reviewer B	Rejected	50	150	200
	Accepted	80	120	200
Total		130	270	400

Cohen's kappa coefficient (Kw)=value; *p*< or *p*>value, concordance =value in percentage.

See Kw on R: https://www.rdocumentation.org/packages/psych/versions/2.1.9/topics/cohen.kappa.

### Appraisal

At the end of the systematic review of documents and data, an evaluation must be carried out to monitor the results obtained [7]. In this sense, guiding questions can be asked:

• Were the search words sufficient to obtain documents that answer the question and objectives?
• Was the spatial and temporal scale sufficient to answer the question?
• Were the documents or data from secondary sources obtained sufficient to answer the question?
• Were the inclusion and exclusion criteria sufficient to obtain the necessary documents?

## SODIP step 2: open-source (software and data) use

The open-source means that the source of the software or data can be modified by an interest group for the purpose of contribution, in addition to being freely accessible to a source of data or software available [33]. Therefore, the use of open-source software is essential, especially due to the reduction or non-existence of costs to manage the data collected in the systematic review.

### Statistical analysis and data visualization

The choice of type of statistical analysis depends on the spatial scale of the systematic review study (Fig. 2). For example, studies that try to identify the trend in a specific ecosystem globally (e.g. “mangrove*”) or locally (“spatial plan*” AND “Brazil”) will use more powerful statistical tools such as machine learning or Geographic Information System (GIS). If the studies are intended to evaluate more specific topics, the analysis will be based on descriptive statistics (Table 11). See Table 9 for choose database.

## SODIP step 3: data visualization and design information

Data visualization is not a simple process, but we can use design and science outreach tools. Therefore, it is essential to direct the visualization and design of systematized data according to the target audience to work as a bridge between society and science [52]. When we manage large amounts of data obtained, for example, from the Scopus or WoS databases, we can facilitate the funneling process by adding systematic review pipelines as a promising tool for optimizing and speeding up the performance of systematic reviews. In addition, Artificial Intelligence (AI) and Machine Learning (ML) have enabled the development of Artificial Intelligence-aided pipelines that assist in finding relevant texts for search tasks.

### Data pipeline with ASReview

**Fig. 3:**
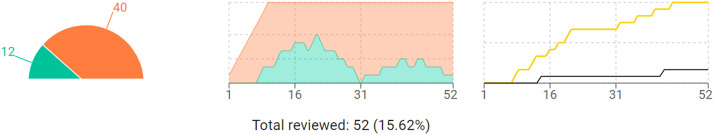
Pipeline example in ASReview.

In addition to being freely accessible, this software has a robust potential for document selection by machine learning [36]. The phases for the selection of documents through this tool are: (i) pre-screening selects the documents, (ii) screening to evaluate the documents by relevance and (iii) post-screening to evaluate the relevance according to the training of the statistician [36]. For example, in one study, more than 10,000 documents were found that were filtered through this tool, excluding 95 % of the documents, leaving for analysis more than 200 documents directly related to the topic addressed [53]. This tool uses Naive Bayes, among other types of statistical analysis, where it is possible to obtain the selected documents (example Fig. 3).

### Visualization of temporal and spatial data

**Fig. 4:**
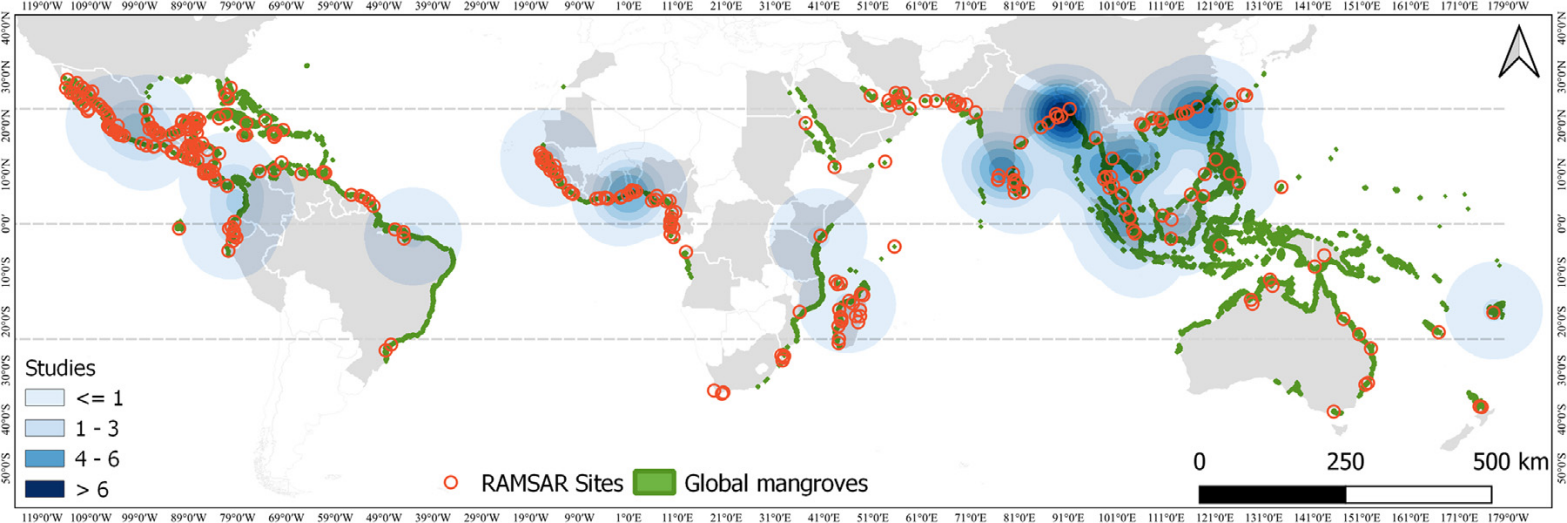
Example of the spatial scale on SDG studies related to mangrove ecosystem [4].

**Fig. 5:**
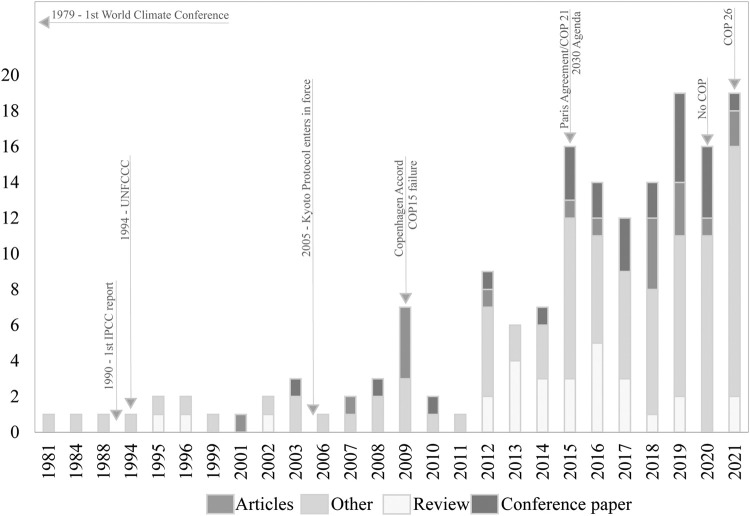
Example of the temporal scale of scientific (WoS) and political (Ecolex) documents. Search words: “mangrove*” AND “climate chang*”.

**Fig. 6:**
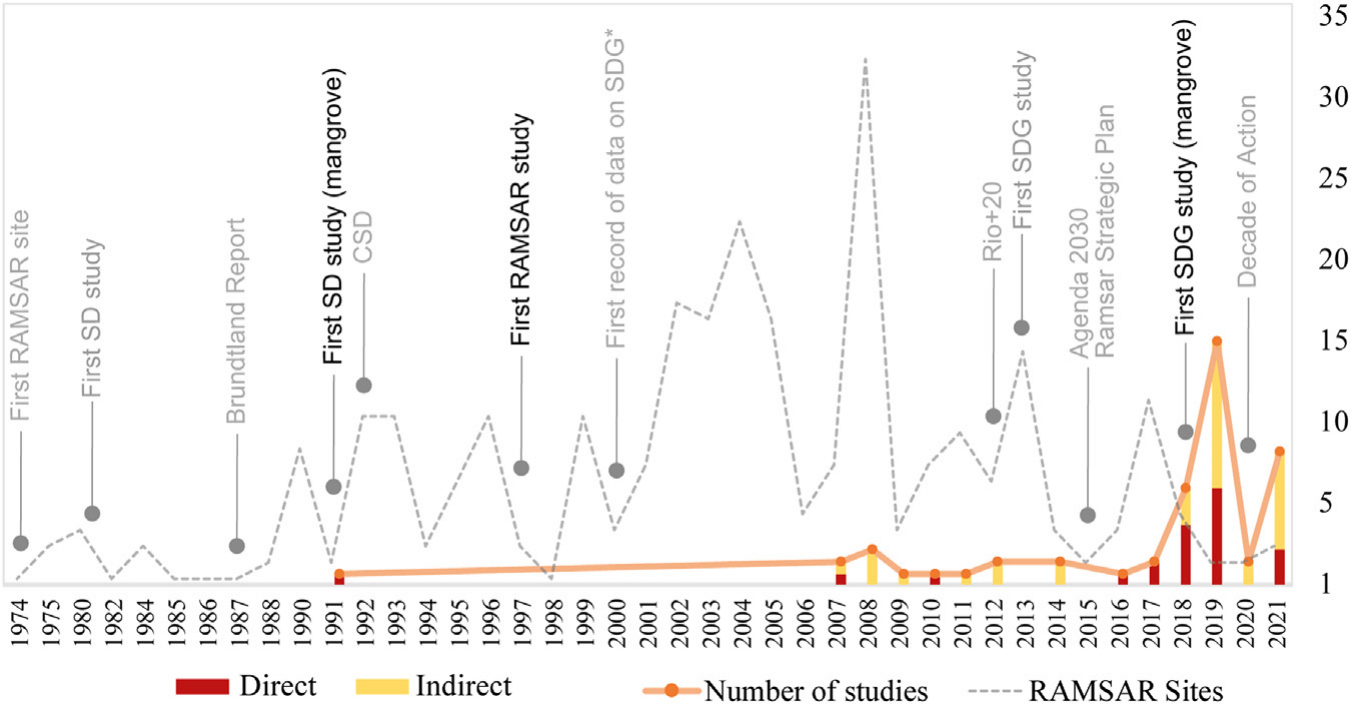
Example of the temporal scale with Important dates [4].

**Fig. 7:**
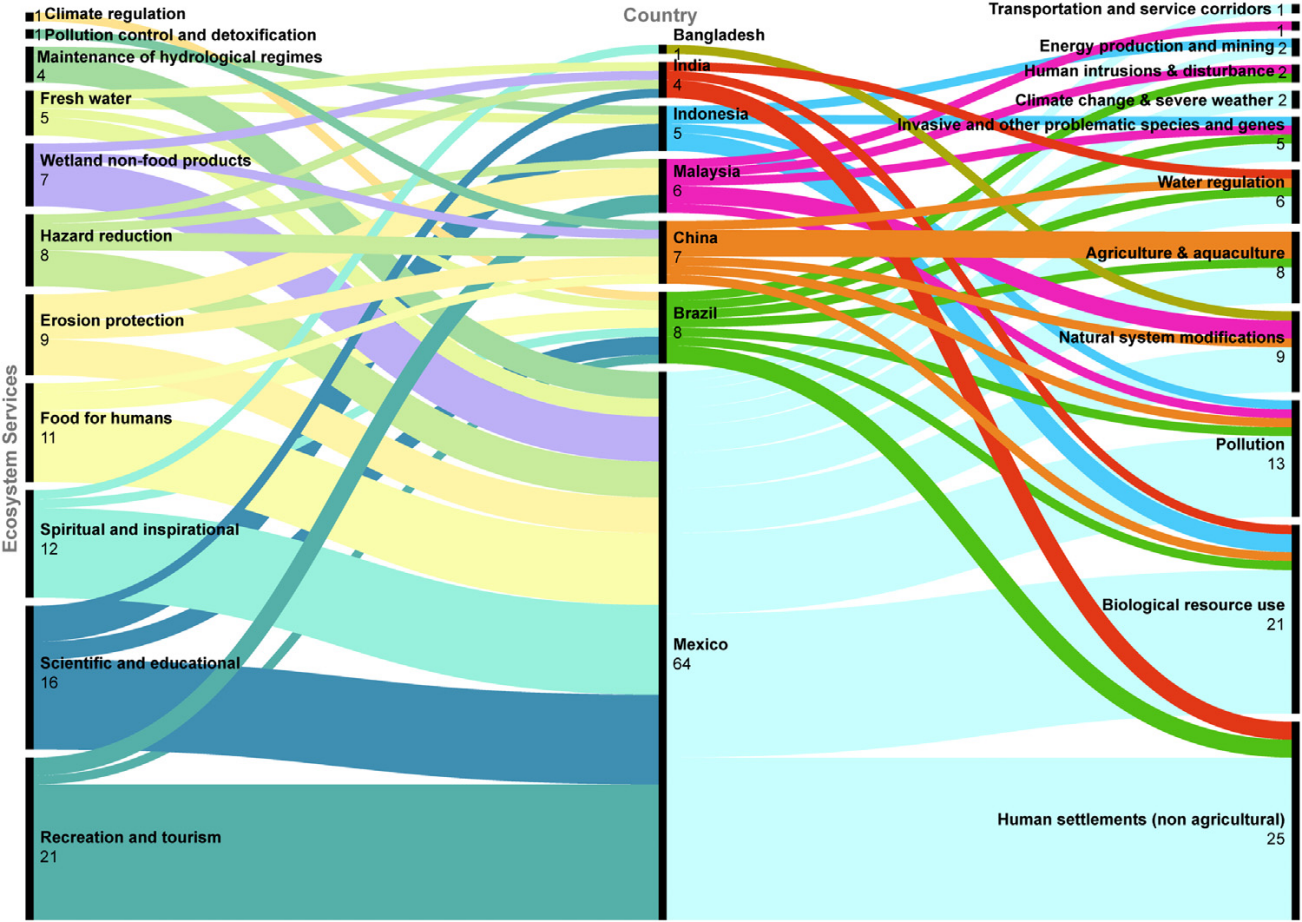
Data visualization with Rawgraphs [4].

**Fig. 8:**
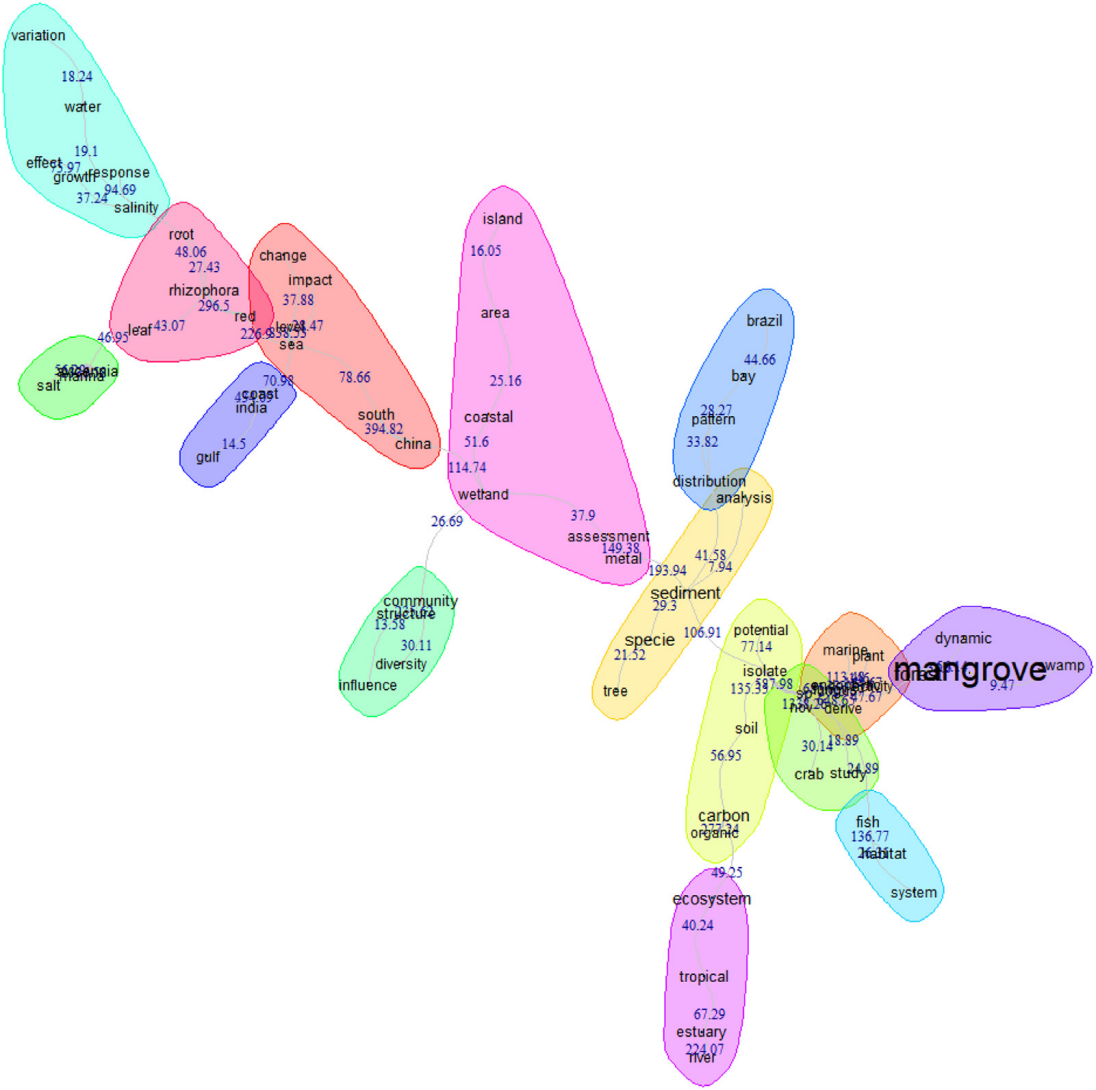
Example of similarity analysis. Search word “mangrove*”.

**Fig. 9:**
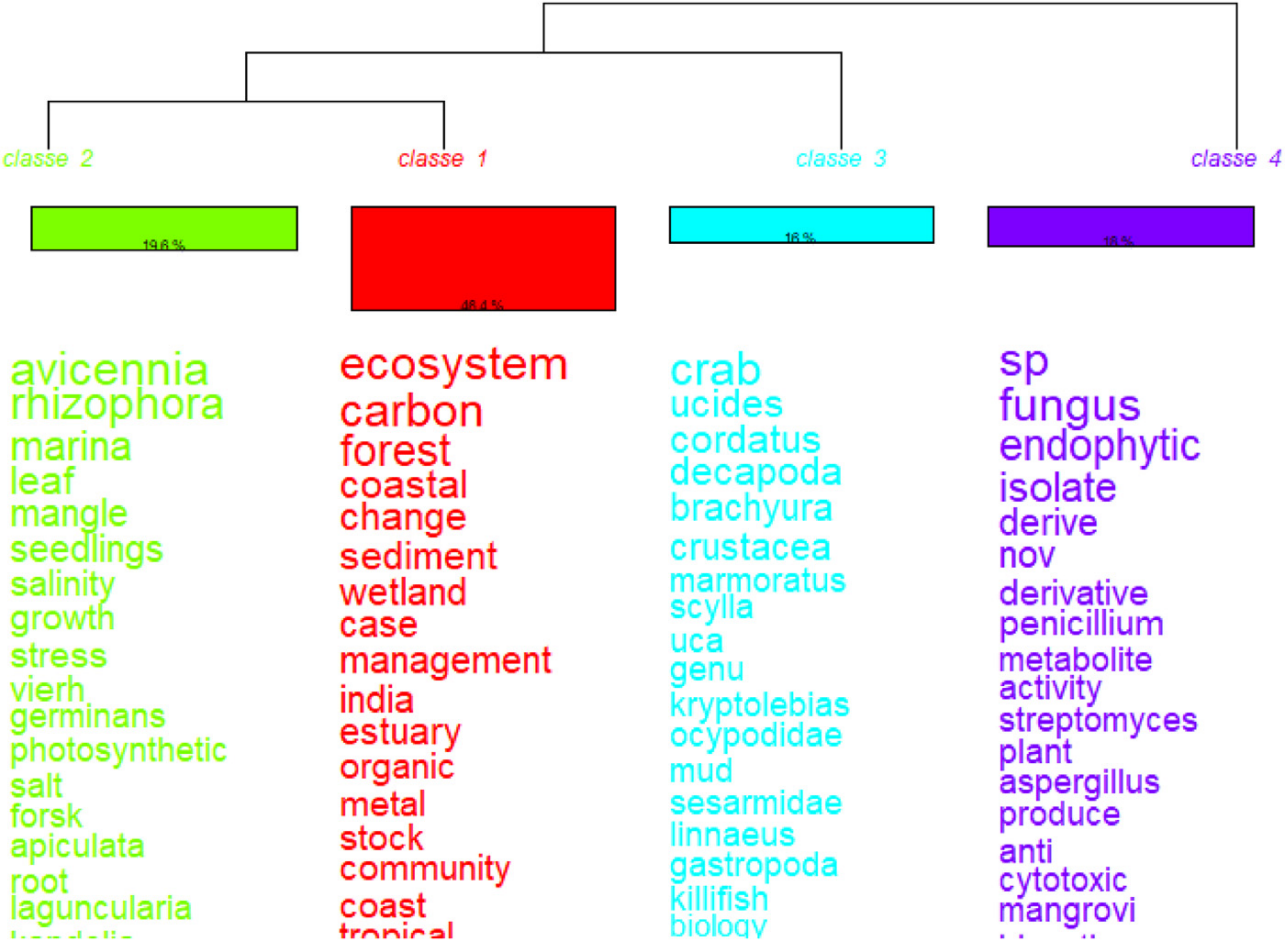
Example of cluster Reinert Method. Search word “mangrove*”.

**Fig. 10:**
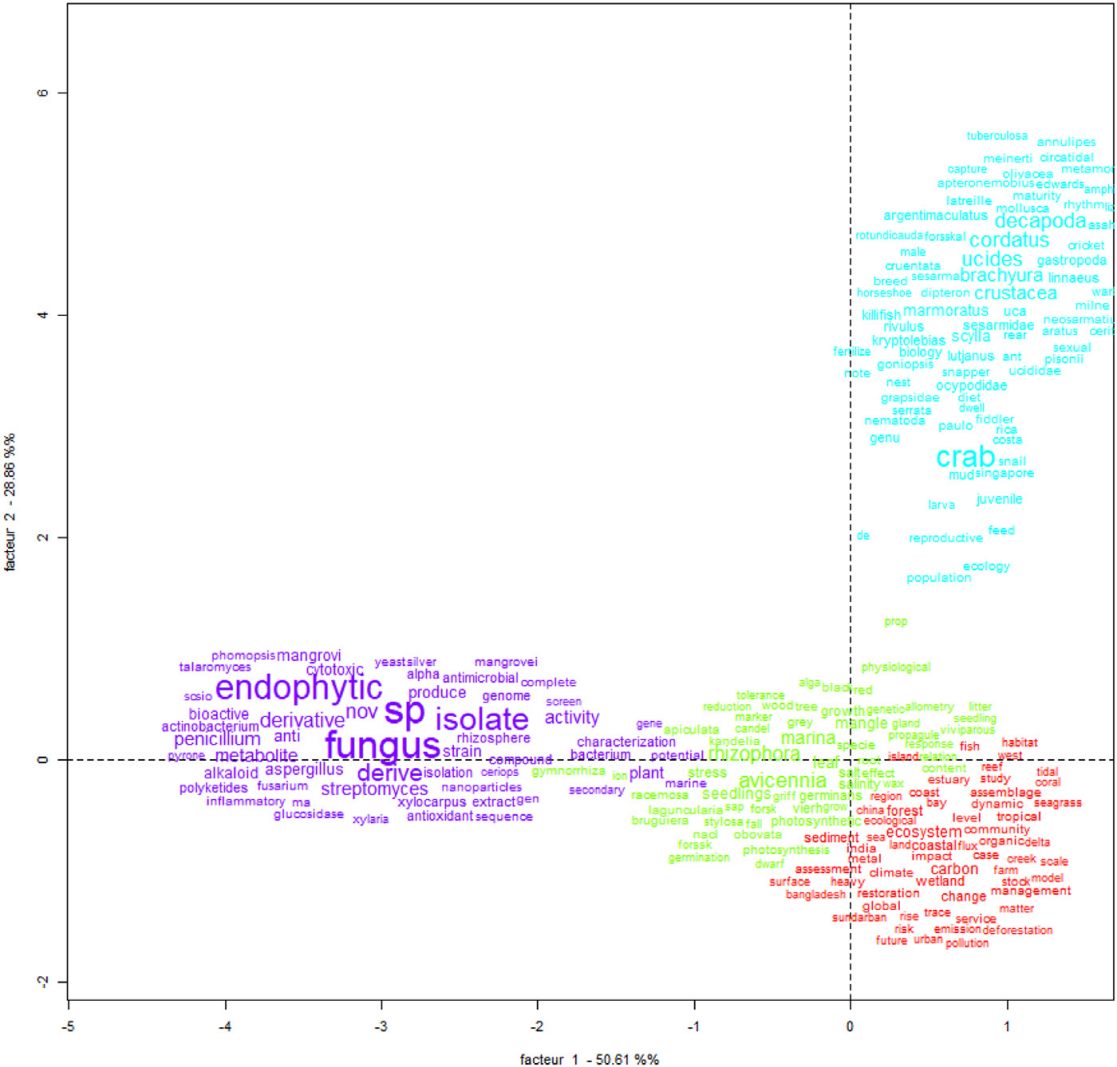
Example of Correspondence Factor Analysis. Search word “mangrove*”.

The information obtained can be translated into figures that show the spatial scale (e.g., maps with georeferenced data) and the temporal scale (e.g., serial figures). For spatial data, a heat map (Kernel map) created by using QGis can be used (Fig. 4), while temporal data can be presented through a timeline (Fig. 5, Fig. 6). Data can be visualized using design such as RawGraphs without needing to know programming language (Fig. 7). The information must have a statistical basis and for this, qualitative analysis tools such as Voyant, for creating a word cloud, or Iramuteq, for similarity analysis (Fig. 8), Reinert Method clusters (Fig. 9), and Correspondence Factor Analysis (Fig. 10). Table 11 presents more open-source tools.

### Collaborative information libraries

Review studies generate a dataset and can be made available in collaborative libraries through open-source tools such as Mendeley and Zotero [54,55]. Likewise, funneled data can be made available through repositories such as Mendeley Data, as it can be the basis for preparing other articles [56]. See the example of a collaborative library about mangroves that has been developed (https://tinyurl.com/mangrovecollaborative).

## SODIP step 4: identifying gaps, challenges and trends

In most reviews we find qualitative data that needs to be analyzed, for example, the sections of articles selected from the Scopus and WoS databases. Sections such as the title, keywords and summary offer information necessary to carry out a theoretical analysis and a scientific diagnosis of the main topics covered in a given type of study. At the same time, there are many qualitative data analysis tools, most of which are paid software, such as NVivo and MAXQDA. Iramuteq, in addition to being free access software, is a very powerful tool for qualitative analysis, serving as support for identifying gaps, challenges, and trends.

### Iramuteq

**Fig. 11:**
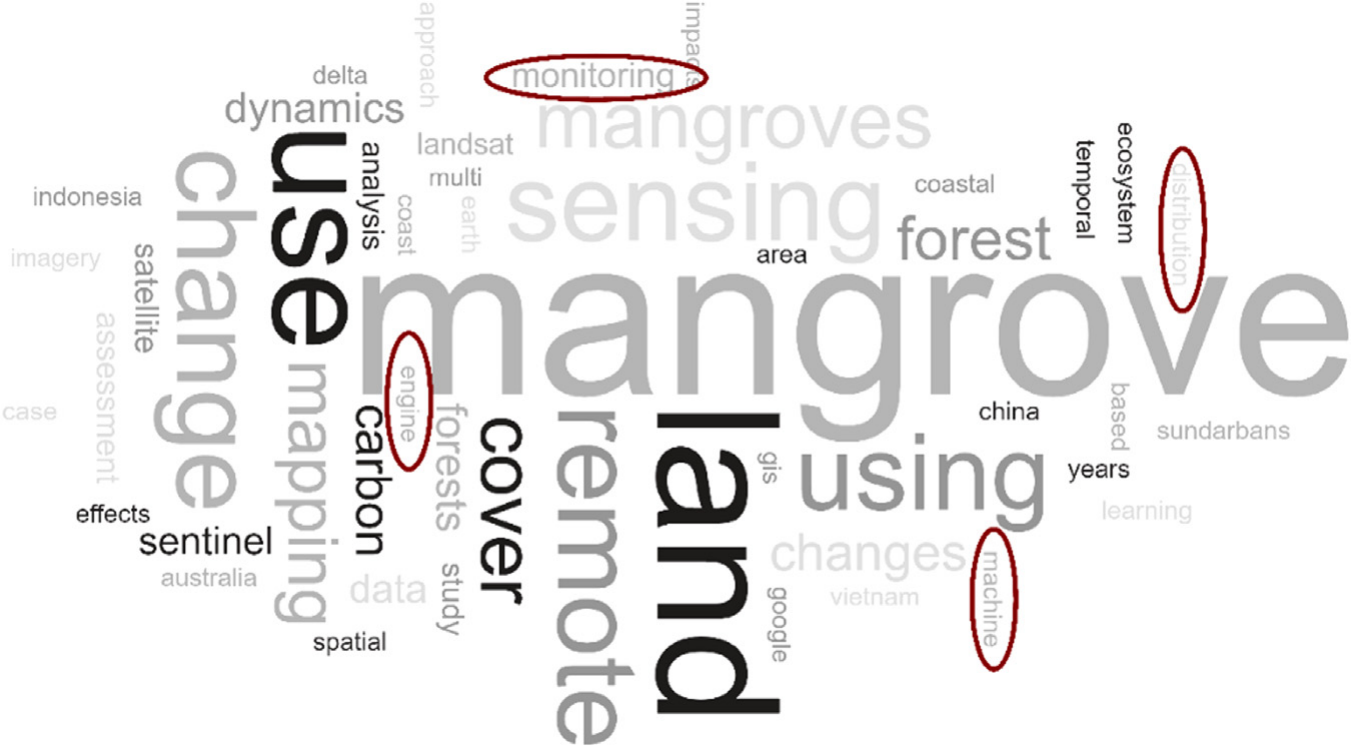
Example of word cloud to identify most frequent topics. Search word “mangrove*”.

**Fig. 12:**
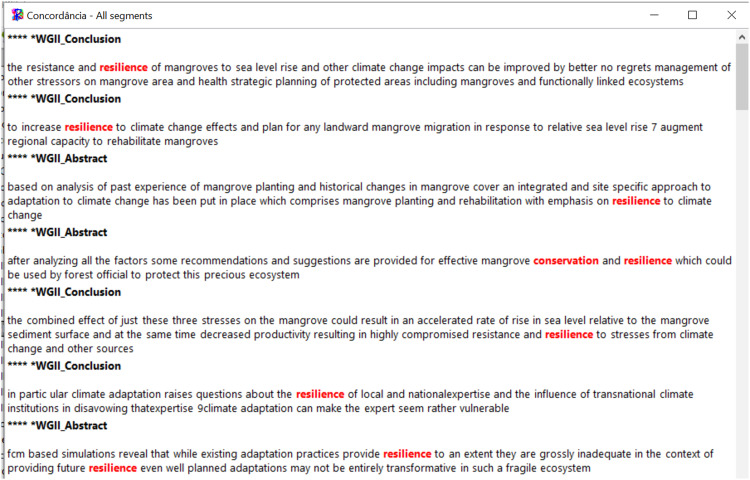
Example co-occurrence of the words by document sections. Search word “mangrove*” and “climate chang*”.

**Fig. 13:**
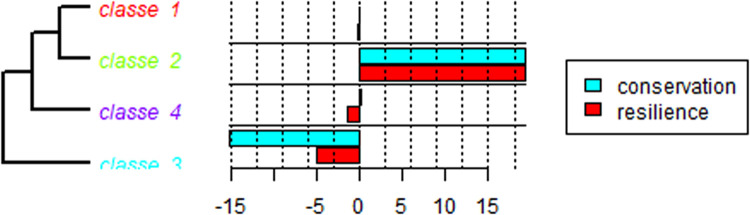
Statistical graphs by class. Search words “mangrove*” and “climate change*”.

**Fig. 14:**
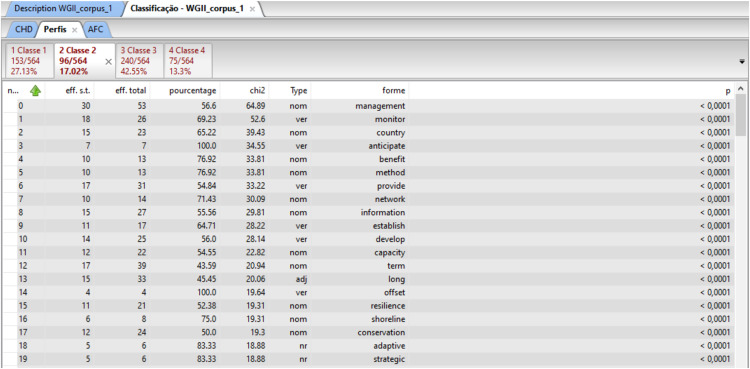
Statistical analyses by class. Search words “mangrove*” and “climate change*”.

This software is based on the R language and can be used to visualize and analyze qualitative data through several analyses: (i) word cloud that group and organize words based on their frequency [57] (Fig. 11), (ii) similarity analysis (Fig. 8), (iii) cluster Reinert Method (Fig. 9), (iv) Prototypical analysis and (v) Correspondence Factor Analysis (Fig. 10). The word cloud is supported to identify the most frequently studied topics in the documents, from this automated identification we can define topics for the review study. See more examples of the Similarity Analysis and Descending Hierarchical Analysis (DHA) in the article “Systematic Review of Spatial Planning and Marine Protected Areas: A Brazilian Perspective” [14]. Similarly, this tool can help in the document content analysis process through the co-occurrence of the words classified by the document sections (Fig. 12) and through statistical graphs from the analysis of the textual corpus (Fig. 13, Fig. 14).

### Decision making when choosing the tool

**Fig. 15:**
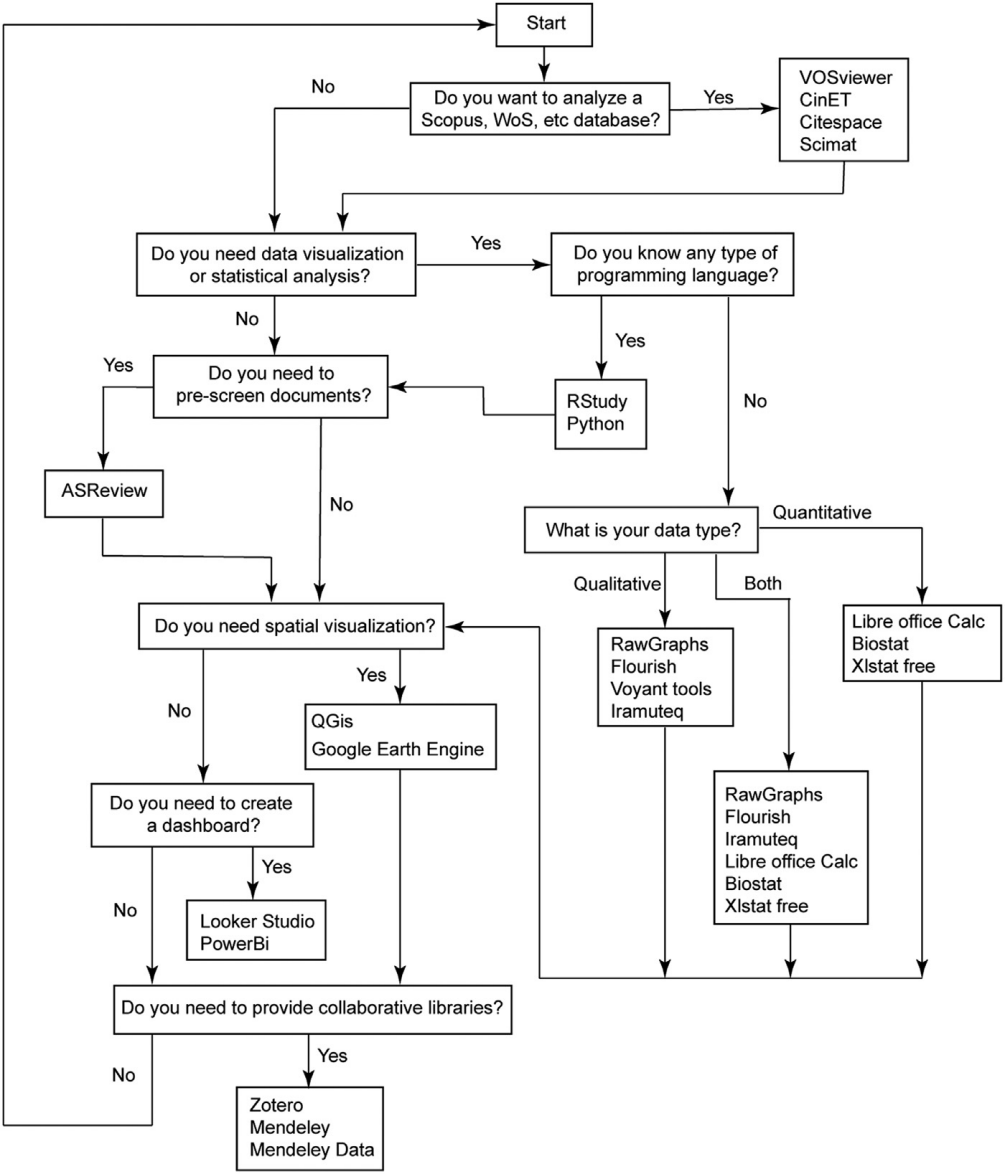
Decision-making flowchart for data visualization.

The entire systematic review process is extremely relevant for proposing a framework. And to facilitate decision-making when choosing one of the tools mentioned in this methodology, a decision flowchart was created according to the type of data obtained (Fig. 15).

## SODIP step 5: propose a conceptual and theoretical framework

The theory is the set of elements (e.g. concepts) that are interconnected and aim to explain and predict from the modelling of a framework [58]. The theory allows applying the key elements in practice, in addition to promoting questioning to generate more research and fill gaps contributing to the construction of theories [58]. There are two definitions to be considered when choosing between conceptual or theoretical framework proposals from the systematic review:

a. *Theoretical framework*: it is the theoretical framework (a mother theory) chosen by researchers to guide the topic addressed, that is, it is epistemology, the explanation of the same theory to address a problem [58]. For example, common goods under the tragedy of commons theory [59].
b. *Conceptual framework*: it is the synthesis of several theories and different perspectives on the topic addressed [58]. For example, goods in common use from a perspective of the tragedy of the commons [59] and Socioecological Systems (SES) [60].

**Fig. 16:**
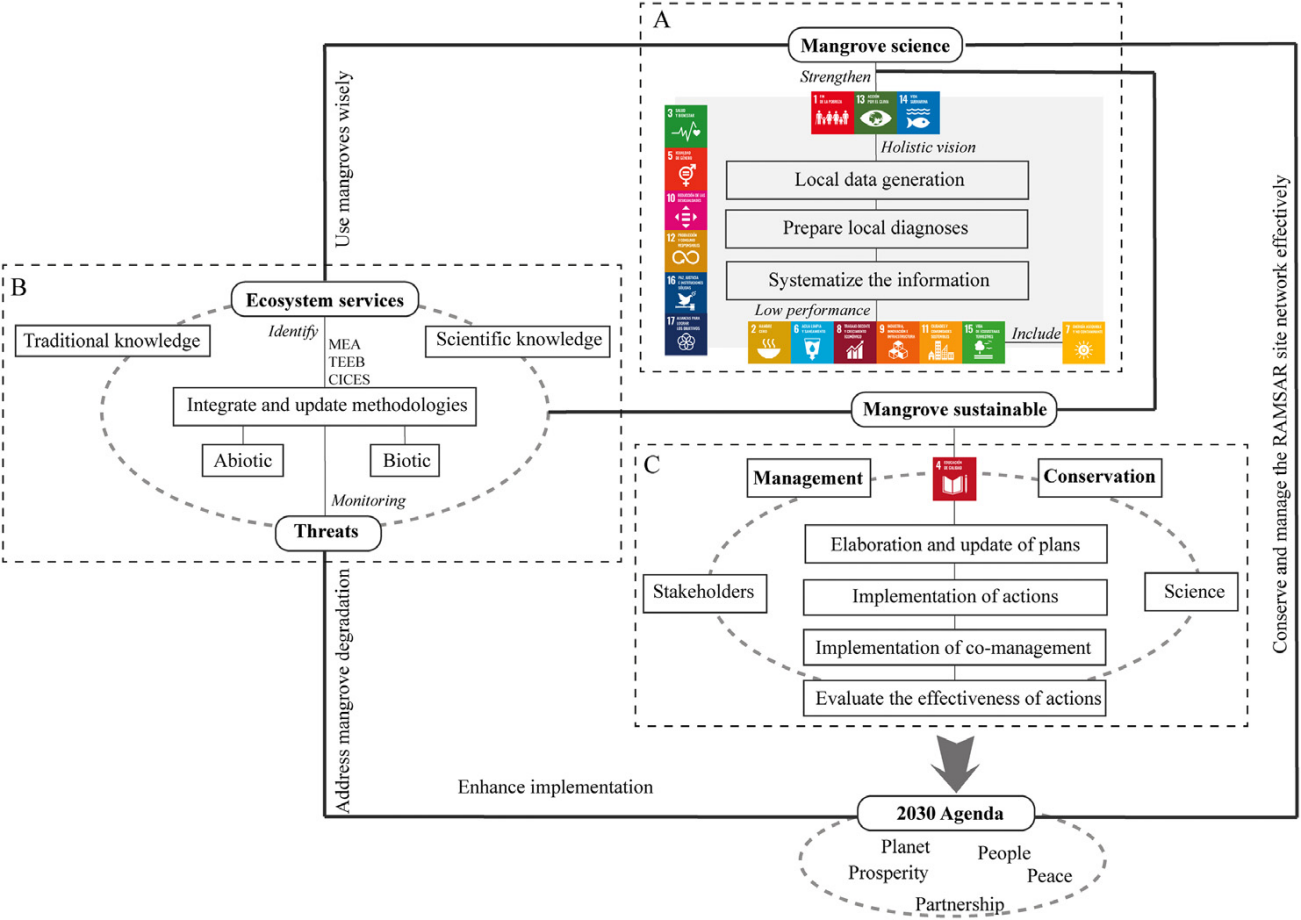
Conceptual framework of mangrove and Sustainable Development Goals (SDGs) [4].

In other words, the conceptual framework is a deeper and more complex study than the theoretical framework, as it holistically uses various theories and perspectives. The purpose of both is to explain a question and propose ways to fill the gaps in the knowledge of the topic addressed (see examples in Table 13). The conceptual and theoretical frameworks can also be proposed following the PICO framework [61] (Fig. 16). Here are some steps to formulate conceptual and theoretical frameworks [58]:

1. Synthesize concepts and perspectives through various sources: obtaining information from systematic review studies, meta-analysis, bibliometrics, scientific document databases, legislation documents and other data in open-access databases.
2. Understand the basic concepts to answer the guiding questions, hypotheses and objectives: identify trends and gaps by answering questions such as: What main topics are covered? What are the topics present or absent in the theory of the topic addressed? What is the spatial scale of the studies or data found? What is the time scale of the studies or data found? Why are studies missing in a period?
3. Include transdisciplinary perspectives to answer a question: What needs to be improved theoretically or conceptually?
4. Propose new ways to build the science of the topic addressed and fill gaps: What are the ways that this theory or concept should direct to new perspectives?

**Table 13:** Examples of studies on conceptual and theoretical frameworks.

Title	Aim	Conceptual	Theoretical
Changing the Conversation about Climate Change: A Theoretical Framework for Place-Based Climate Change Engagement	"In this paper, we present and test a theoretical framework for place-based climate change engagement (…) Our framework is based on place attachment, place-based education, free-choice learning, and norm activation theories." [62]		X
Vulnerability: A generally applicable conceptual framework for climate change research	"This paper presents a generally applicable conceptual framework of vulnerability that combines a nomenclature of vulnerable situations and terminology of vulnerability concepts based on the distinction of four fundamental groups of vulnerability factors. This conceptual framework is applied to characterize the vulnerability concepts employed by the main schools of vulnerability research and to review earlier attempts at classifying vulnerability concepts." [63]	X	
The theoretical framework for evaluation of cross-cultural training effectiveness	“This review shows a trend toward the broadening of evaluation research, using many more dependent variables, with measurements obtained from many kinds of people.” [64]		X
Ramsar Wetlands of International Importance–Improving Conservation Outcomes	“We provide a perspective on achieving these goals and targets, focusing on two key objectives: (1) identification of biases in the current global distribution of the Ramsar Site Network and (2) a conceptual adaptive management framework, linking maintenance of ecosystem dynamics with drivers of change.” [65]	X	
A conceptual framework for systematic reviews of research in educational leadership and management	“The purpose of this paper is to present a framework for scholars carrying out reviews of research that meet international standards for publication.” [66]	X	
The Internet of Things: Review and theoretical framework	“This study includes a systematic review and synthesis of IoT related literature and the development of a theoretical framework and conceptual model.” [67]		X
Integrating a conceptual framework for the sustainable development goals in the mangrove ecosystem: Asystematic review	“A conceptual framework for the assessment of SDGs in relation to the mangrove ecosystem is needed to fulfil the Ramsar Sites Strategic Plan and the 2030 Agenda based on their ecosystem services in order to address the identified threats.”	X

**Fig. 17:**
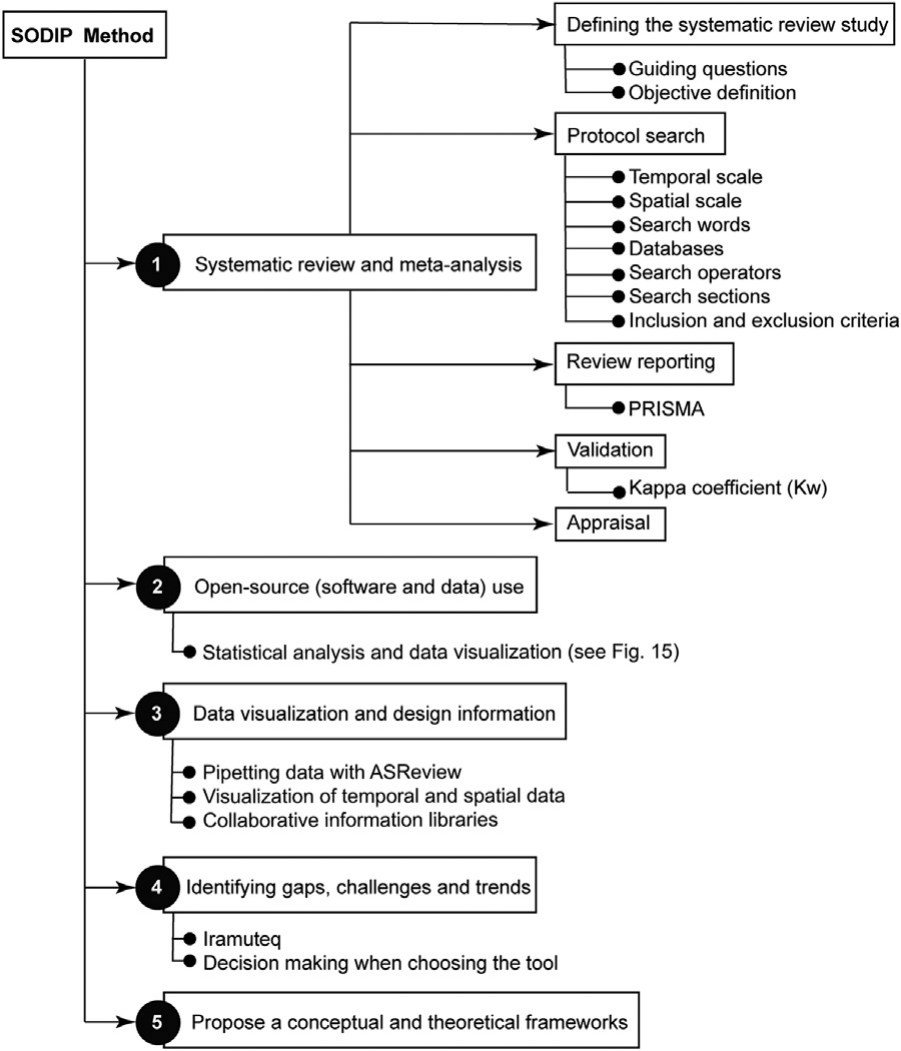
Flowchart of the SODIP methodology.

Fig. 17 presents an overview of the proposed methodology.

## Glossary

***Bibliometry:*** The term bibliometrics was used for the first time in 1969 and has the advantage of studying geographic distribution, word frequency, and citations, among other relevant aspects of scientific documents [68]. Bibliometrics is a method that statistically measures the relationship of scientific documents [1,69]. The main indicators are i) quantity of measuring the productivity of the research group, ii) performance indicators of the quality of the journal, authors and other fields of research and iii) structural indicators of the connections between the previous themes [1,2]. Bibliometrics identifies theoretical trends in a topic addressed and explores future theoretical directions [70], in addition to using big data to analyze these trends and identify gaps in knowledge. In addition, bibliometric studies need data encoded in databases [15].
**Database:** the systematized set of data (information) stored [71] (e.g. historical data stored in the Worldbank database).
**Databank:** a large quantity of data on a particular topic [71] (e.g. historical data on SDG at the UN).
**Data set:** a collection of data [71] (e.g. Earth Observation Data the NASA).

## CRediT authorship contribution statement

**Indira A.  L. Eyzaguirre:** Conceptualization, Methodology, Data curation, Visualization, Writing – original draft, Writing – review & editing. **Marcus E.  B. Fernandes:** Supervision, Writing – review & editing.

## Declaration of competing interest

None. The authors declare that they have no known competing financial interests or personal relationships that could have appeared to influence the work reported in this paper.

## Data Availability

We provide the supplementary file.
